# Programmed cell death ligand 1 (PD-L1, CD274) in cholangiocarcinoma – correlation with clinicopathological data and comparison of antibodies

**DOI:** 10.1186/s12885-018-5254-0

**Published:** 2019-01-15

**Authors:** Mark Kriegsmann, Stephanie Roessler, Katharina Kriegsmann, Marcus Renner, Rémi Longuespée, Thomas Albrecht, Moritz Loeffler, Stephan Singer, Arianeb Mehrabi, Monika Nadja Vogel, Anita Pathil, Bruno Köhler, Christoph Springfeld, Christian Rupp, Karl Heinz Weiss, Benjamin Goeppert

**Affiliations:** 1 Institute of Pathology, University Heidelberg, Im Neuenheimer Feld 224, Heidelberg, Germany; 2 Department of Rheumatology, Oncology and Hematology, University of Heidelberg, Heidelberg, Germany; 3 Department of General Visceral and Transplantation Surgery, University Hospital Heidelberg, Im Neuenheimer Feld 110, Heidelberg, Germany; 4 Diagnostic and Interventional Radiology, Thoraxklinik at University Hospital of Heidelberg, Heidelberg, Germany; 5 Department of Internal Medicine IV, Gastroenterology and Hepatology, University Hospital Heidelberg, Im Neuenheimer Feld 410, Heidelberg, Germany; 6 Department of Medical Oncology, University Hospital Heidelberg, National Center for Tumor Diseases, Heidelberg, Germany; 7 Liver Cancer Center Heidelberg (LCCH), Heidelberg, Germany

**Keywords:** PD-L1, Cholangiocarcinoma, CD274, 28–8, SP142, SP263

## Abstract

**Background:**

Cholangiocarcinoma (CCA) may arise in the intra- or extrahepatic biliary tract and is associated with a poor prognosis. Despite recent advances, to date there is still no established targeted therapeutic approach available. Non-surgical therapeutic agents are urgently needed, as most patients are non-eligible to surgical resection. Anti-PD-L1 therapy prevents cancer cells from evading the immune system and has emerged as a new treatment option in several cancer entities. Recently, PD-L1 expression has been analyzed in comparably small CCA patient cohorts. However, a systematic validation of different PD-L1 antibodies has not been performed in CCA so far.

**Methods:**

We stained a tissue microarray consisting of 170 patients, including 72 intrahepatic cholangiocarcinomas (iCCAs), 57 perihilar cholangiocarcinomas (pCCAs) and 41 distal cholangiocarcinomas (dCCAs) by immunohistochemistry and evaluated PD-L1 positivity in tumor and stromal cells. We analyzed three different PD-L1 antibodies (clones 28–8, SP142, and SP263) that are frequently used and recommended for predictive diagnostic testing in other cancer types.

**Results:**

For PD-L1 antibody clone SP263, 5% of iCCAs, 4% of pCCAs and 3% of dCCAs exhibited PD-L1 expression on tumor cells, thereby showing the highest frequencies of PD-L1 positivity. Accordingly, highest PD-L1 positivity rates of stromal cells with 31% in iCCA, 40% in pCCA and 61% in dCCA were detected for clone SP263. Agreement of PD-L1 positivity in tumor cells was moderate for clone 28–8 and SP263 (*κ* = 0.44) and poor between 28-8 and SP142 (*κ* = 0.13), as well as  SP142 and SP263 (*κ* = 0.11), respectively. Statistical analyses of PD-L1 expression (clone SP263) on tumor cells with clinicopathological data revealed a positive correlation with shortened overall survival in CCA patients.

**Conclusions:**

Selection of appropriate PD-L1 antibodies and careful evaluation of immunohistochemical staining patterns have a significant impact on PD-L1 testing in CCA. Clinical trials are necessary to investigate the putative beneficial effects of PD-L1 targeted immunotherapy in CCA patients.

**Electronic supplementary material:**

The online version of this article (10.1186/s12885-018-5254-0) contains supplementary material, which is available to authorized users.

## Background

Recently, a novel class of small molecules was developed to inhibit the association of programmed cell death ligand 1 (PD-L1) with its receptor programmed cell death protein 1 (PD-1). PD-L1 is expressed on tumor cells and interacts with PD-1 on cytotoxic T-cells, leading to reduced T-cell function and thereby lower anti-tumor activity of the host’s immune system [1]. Thus, PD-L1 inhibitors prevent cancer cells from evading the immune system. These agents have been found effective in melanoma [2–4], non-small cell lung cancer [4–6], renal cell carcinoma [4, 7] and urothelial carcinoma [8], and were consequently approved by the Food and Drug Administration for these cancer types [9].

Standardized interpretation criteria for predictive PD-L1 testing are still matter of debate, and data of comprehensive and well-characterized cohorts are not available for rare cancer types [10], including cholangiocarcinoma (CCA), which is a heterogeneous group of malignancies that can emerge at any location in the biliary tree, from the smallest intrahepatic  bile ducts (Canaliculi biliferi) to the distal choledochal duct. According to the anatomical location, CCAs are subclassified into intrahepatic (iCCA), perihilar (pCCA), and distal (dCCA) tumors [11]. Although CCAs and biliary tract cancers have often been treated as one tumor type in the past [12], it is nowadays widely accepted that CCAs, and in particular intrahepatic and extrahepatic subtypes display significant clinical, biological, and therefore therapeutically relevant differences, leading to the consensus that all subtypes should be regarded differentially [13, 14]. Clinical studies providing adequate evidence on the efficacy of PD-L1 therapy in CCA are not yet available. However, based on the success in other cancer entities, these agents hold promise in the therapy of CCA [15]. At present, one of the main challenges is to select patient subgroups that are likely to benefit from anti-PD-L1 therapy. Therefore, upfront immunohistochemical staining with antibodies against PD-L1 is recommended to differentiate responders from non-responders [16]. Moreover, PD-L1 expression has been shown to correlate with a worse outcome in a meta-analysis including various cancer types and more than 16,000 patient samples, but CCA was not included [17].

Regarding the existing studies, a comprehensive view of PD-L1 expression in CCA including all subtypes and correlation with clinicopathological data is not available to date. Moreover, it has become evident that PD-L1 testing is highly dependent on usage of stringent evaluation criteria and the selection of appropriate PD-L1 antibody clones [18].

In this study, we analyzed PD-L1 expression on tissue samples of 170 CCA patients using tissue microarrays (TMAs) to provide a solid database of PD-L1 expression in Western CCA and its subtypes. Additionally, we compared the staining results of three different PD-L1 antibodies and tested their level of agreement. Furthermore, we correlated the results of the antibody with the highest positivity rates (clone SP263) with clinicopathological variables including overall survival of CCA patients.

## Methods

### Cholangiocarcinoma cohort characteristics

Formalin-fixed and paraffin embedded (FFPE) specimens resected from 1995 to 2010 at Heidelberg University Hospital were extracted from the archive of the Institute of Pathology, Heidelberg University, with the support of the tissue bank of the National Center for Tumor Diseases (NCT; project # 2116). Only resection cases were included. None of the patients received radio- or chemotherapy prior to surgery. Diagnoses were made according to the World Health Organization (WHO) classification of Tumors of the Digestive System 2010. Tumors were restaged according to the 8th UICC/AJCC TNM Classification Manual by two experienced pathologists. The CCA cohort consisted only of adenocarcinomas, including all histological variants. The cohort consisted of 170 patients including 72 iCCAs, 57 pCCAs, and 41 dCCAs. Ampullary tumors were not included in this study, as they are often of intestinal histologic differentiation, and represent a different tumor entity both, clinically and biologically. Survival data was available for 141 patients. Detailed clinicopathological data is displayed in Table 1.

**Table 1:** Clinicopathological characteristics of the cholangiocarcinoma cohort

		All cases	PD-L1 (< 1%)	PD-L1 (≥1%)	*p*-value
	Number (%)	170 (100)	151 (89)	19 (11)	
Age	Range (years)	31–91	31–91	34–78	0.886 *
	Median (years)	63	63	66	
	<Median (total)	85 (50)	76 (45)	9 (5)	1.000 **
	>Median (total)	85 (50)	75 (44)	10 (6)	
Sex	male	109 (64)	94 (55)	15 (9)	0.206 **
	female	61 (36)	57 (34)	4 (2)	
CCA subgroups	iCCA	72 (42)	64 (38)	8 (5)	0.967 ***
	pCCA	57 (34)	51 (30)	6 (4)	
	dCCA	41 (24)	36 (21)	5 (3)	
UICC stage^#^	UICC 1	6 (4)	6 (4)	0 (0)	0.481 ***
	UICC 2	62 (36)	56 (33)	6 (4)	
	UICC 3	45 (26)	37 (22)	8 (5)	
	UICC 4	16 (9)	14 (8)	2 (1)	
	NA	41 (24)	38 (22)	3 (2)	
pT	T1	21 (12)	19 (11)	2 (1)	0.183 ***
	T2	91 (54)	83 (49)	8 (5)	
	T3	44 (26)	39 (23)	5 (3)	
	T4	14 (8)	10 (6)	4 (2)	
pN	N0	53 (31)	49 (29)	4 (2)	0.267 **
	N1	72 (42)	61 (36)	11 (6)	
	NX	45 (26)	41 (24)	4 (2)	
M	M0	153 (90)	136 (80)	17 (10)	1.000 **
	M1	16 (9)	15 (9)	1 (1)	
	NA	1 (1)	0 (0)	1 (1)	
G	G1	8 (5)	6 (4)	2 (1)	0.135 ***
	G2	121 (71)	111 (65)	10 (6)	
	G3	41 (24)	34 (20)	7 (4)	
L	L0	86 (51)	78 (46)	8 (5)	0.474 **
	L1	84 (49)	73 (43)	11 (6)	
V	V0	125 (74)	111 (65)	14 (8)	1.000 **
	V1	45 (26)	40 (24)	5 (3)	
Pn	Pn0	97 (57)	87 (51)	10 (6)	0.807 **
	Pn1	73 (43)	64 (38)	9 (5)	
R	R0	76 (45)	63 (37)	13 (8)	0.109 ***
	R1	56 (33)	53 (31)	3 (2)	
	R2	12 (7)	11 (6)	1 (1)	
	Rx	26 (15)	24 (14)	2 (1)	

* Mann-Whitney U-test; ** Fisher’s exact test; *** Chi-square test

# Cases with pNX had no lymph nodes resected, therefore, UICC status could not be assessed

*iCCA* intrahepatic cholangiocarcinoma, *pCCA* perihilar cholangiocarcinoma, *dCCA* distal cholangiocarcinoma, *NA* not available

### Tissue microarray construction

From all 170 CCA FFPE tissue blocks, 3 μm sections were cut and stained with H&E. Representative areas were marked by two experienced pathologists (BG and SS). In each case, tumor tissue cores (1.0 mm diameter) from the selected representative tumor areas were punched out of the sample tissue blocks and embedded into a new paraffin array block using a tissue microarrayer (Beecher Instruments, Woodland, CA, USA). On-slide control tissues (tonsil and gallbladder) were used.

### PD-L1 immunohistochemistry

PD-L1 expression analysis was performed using three different antibodies against PD-L1 (clone 28–8 (Abcam plc, Cambridge, UK), clone SP142 (Linaris GmbH, Dossenheim, Germany), and clone SP263 (Roche AG, Rotkreuz, Switzerland)). In brief, 3 μm sections of the TMA were deparaffinized, pre-treated with an antigen retrieval buffer (Tris/Borat/EDTA, pH 8.4; Ventana, Roche) and stained using an automated device (Ventana Benchmark Ultra, Roche). Dilutions were as follows: 1:100 for antibody 28–8, 1:25 for antibody SP142, and a ready-to-use kit for antibody SP263. Tumor cells and surrounding tumor stroma, including inflammatory infiltrates, were scored separately. The number of cells showing membranous staining was evaluated in percentage. According to the German consensus recommendations for immunohistochemical evaluation of PD-L1, any positivity was defined as ≥1% of positive cells having at least weak membranous staining [10]. Tumor cells with pure cytoplasmic staining were scored negatively. Figures were created using Inkscape (v.0.91, Free Software Foundation, Inc., Boston, USA) and R (www.r-project.org, v.3.2.5, Free Software Foundation).

### Statistical analyses

Equally distributed continuous variables were analyzed by Student’s t-test and unequally distributed variables by Wilcoxon-Mann-Whitney test. Distribution data were analyzed by Fisher’s exact test or χ2, where appropriate. Cohen’s statistic was performed to test for agreement. *κ*-values from 0.00 to 0.20 were considered as slight agreement, from 0.21 to 0.40 fair agreement, from 0.41 to 0.60 moderate agreement, from 0.61 to 0.80 substantial agreement and from 0.81 to 1.00 almost perfect agreement. OS was analyzed using the Kaplan–Meier method, with a log-rank test to test for significance. All tests were performed in R programming language and R-Studio (v.0.98.507, Affero General Public License, Boston, USA). Plots were created by R-packages: ggplot2 (v. 2.1.0), and survival (v.2.37–7). *P*-values ≤0.05 were considered significant.

## Results

### PD-L1 immunohistochemistry in the cholangiocarcinoma cohort using three different antibodies

**Fig. 1:**
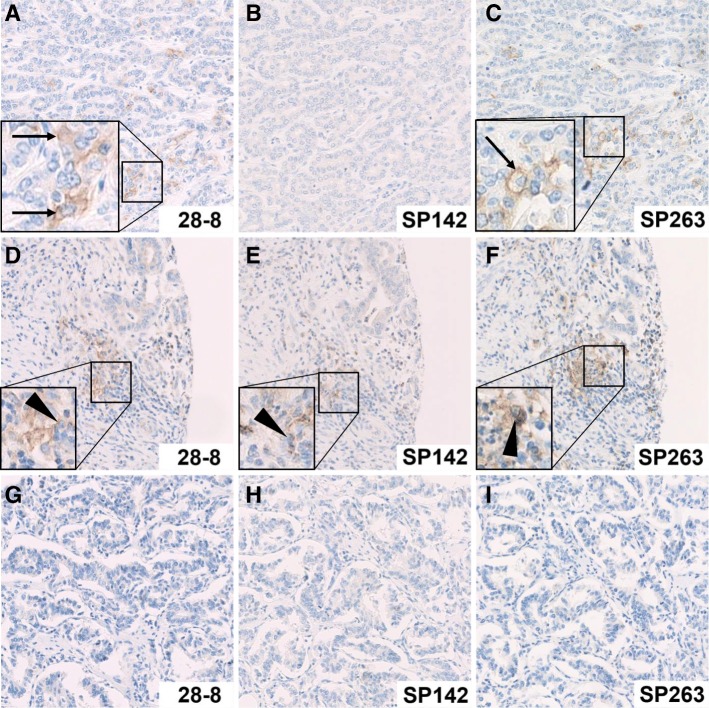
Examples of PD-L1 staining in a representative intrahepatic cholangiocarcinoma. The different staining characteristics of PD-L1 clones 28–8, SP142 and SP263 are displayed. In the first row, tumor cells show membranous positivity of few tumor cells with antibody clones 28–8 (**a**) and SP263 (**c**), but not with clone SP142 (**b**). In the second row, stromal inflammatory cells show membranous PD-L1 expression with all three antibody clones (**d**-**f**), while tumor cells are negative. In the third row, all samples are negative, both in tumor and stromal cells, and with all three PD-L1 antibodies (**g**-**i**). Original magnification: 200x, PD-L1 positive tumor cells are highlighted by black arrows, PD-L1 positive stromal cells are highlighted by black triangles

The complete CCA cohort (*n* = 170) was analyzed three-times independently by PD-L1 immunohistochemistry employing three different PD-L1 antibodies (28–8, SP142 and SP263, Fig. 1). Due to floating or rolling of tissue cores, all three PD-L1 antibody clones could be evaluated in all but one CCA patient (169 out of 170, drop-out rate: < 1%). All three different CCA subtypes, i. e. iCCA, pCCA and dCCA were analyzed separately. PD-L1 antibody displaying the highest positivity rate in CCA was clone SP263 (19/170, 11%, see Table 2). Detailed staining results of clone SP263, including CCA subtype specific evaluation and separated results for tumor and stromal cells, are summarized in Table 2. Generally, more cases showed PD-L1 positivity in stroma than in tumor cells (*p* < 0.01). On-slide control tissues exhibited the following staining pattern: crypt epithelium of tonsils showed strong membranous immunoreactivity and immune cells in germinal centers were weakly positive, as expected; gallbladder epithelium was negative and occasionally immune cells in stromal tissue were positive (Additional file 1: Figure S1).

**Table 2:** PD-L1 expression in cholangiocarcinoma subtypes (tumor and stromal cells; PD-L1 antibody clone: SP263)

Tumor subgroups	Tumor		Stroma	
	Positive	Negative	Positive	Negative
iCCA, *n* = 72				
n (%)	8 (11)	64 (89)	22 (31)	50 (69)
% cells, median (range)	15 (5–30)	n.a.	5 (1–40)	n.a.
pCCA, *n* = 57				
n (%)	6 (11)	51 (89)	23 (40)	34 (60)
% cells, median (range)	10 (5–80)	n.a.	5 (2–30)	n.a.
dCCA, *n* = 41				
n (%)	5 (12)	36 (88)	25 (61)	16 (39)
% cells, median (range)	10 (5–30)	n.a.	5 (1–30)	n.a.

*iCCA* intrahepatic cholangiocarcinoma, *pCCA* perihilar cholangiocarcinoma, *dCCA* distal cholangiocarcinoma, *n.a.* not applicable

### PD-L1 immunohistochemistry in the cholangiocarcinoma cohort and agreement between different PD-L1 antibodies

To test whether the PD-L1 antibodies 28–8, SP142 and SP263 showed similar staining characteristics, agreement between all three clones in tumor and stroma cells was determined. Whereas clone 28–8 and SP263 were positive in a similar number of tumor samples (8 (5%) in iCCA, 6 (4%) in pCCA and 5 (3%) in dCCA), SP142 exhibited tumoral positivity in only two cases. Interestingly, agreement between 28-8 and SP263 in tumor cells was only moderate (*κ* = 0.44), although the amount of positive tumor samples was similar (11% each). Agreement of SP142 and the other two clones was rather poor (*κ* = 0.13 [28-8] and *κ* = 0.11 [SP263]). Overall agreement of the three PD-L1 clones in tumor cells was fair (*κ* = 0.25).

In stroma cells, clones 28–8, SP142 and SP263 were positive in 31, 40 and 61% of cases, respectively. Agreement between the three clones ranged from fair to substantial (*κ* = 0.37 to *κ* = 0.70). A summary for agreement values is provided in Table 3.

**Table 3:**
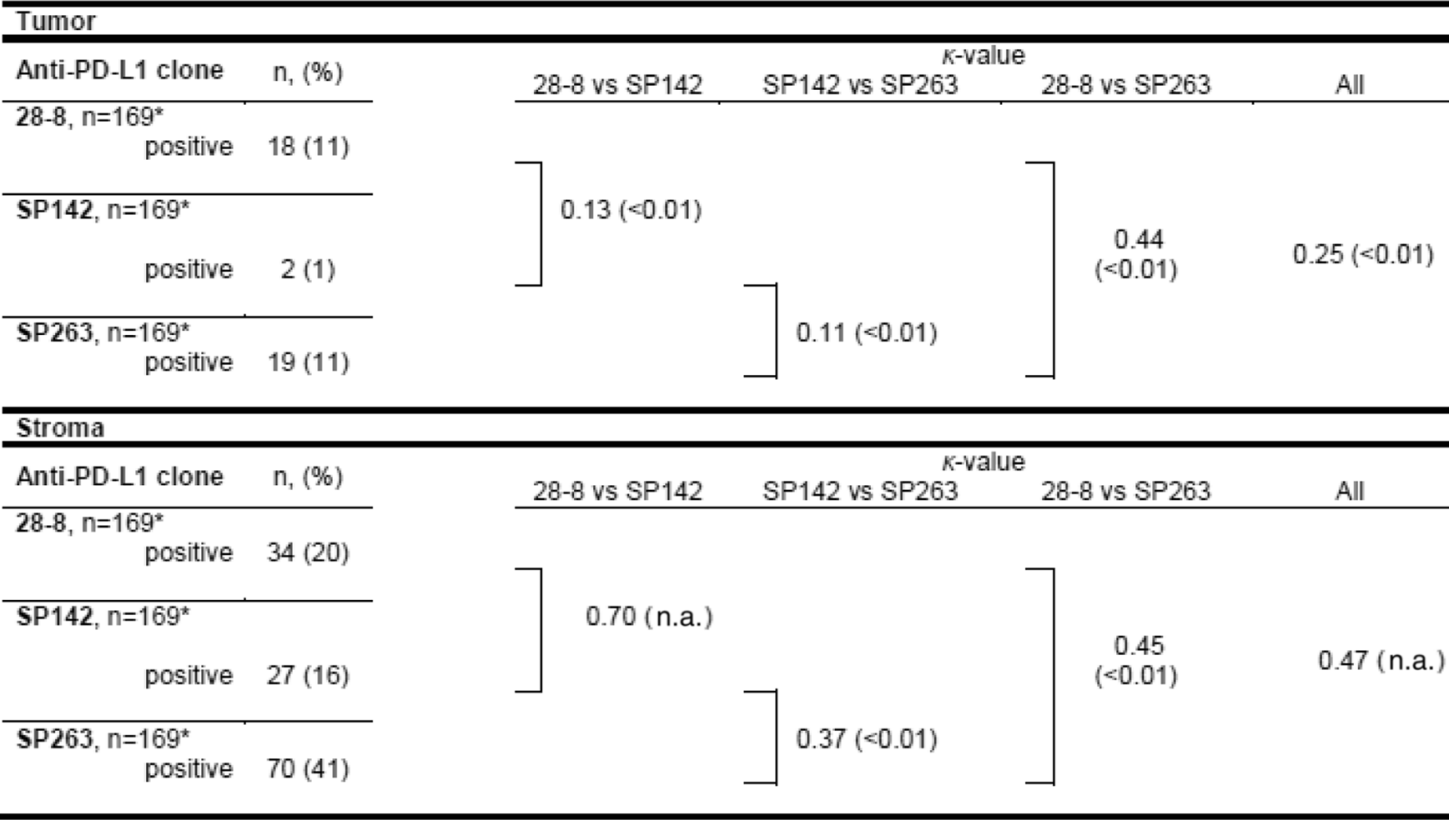
PD-L1 agreement in tumor and stromal cells of the cholangiocarcinoma cohort

*One CCA sample could not be evaluated for all three antibody clones due to technical reasons

### PD-L1 status in correlation with clinicopathological data of the cholangiocarcinoma cohort

**Fig. 2:**
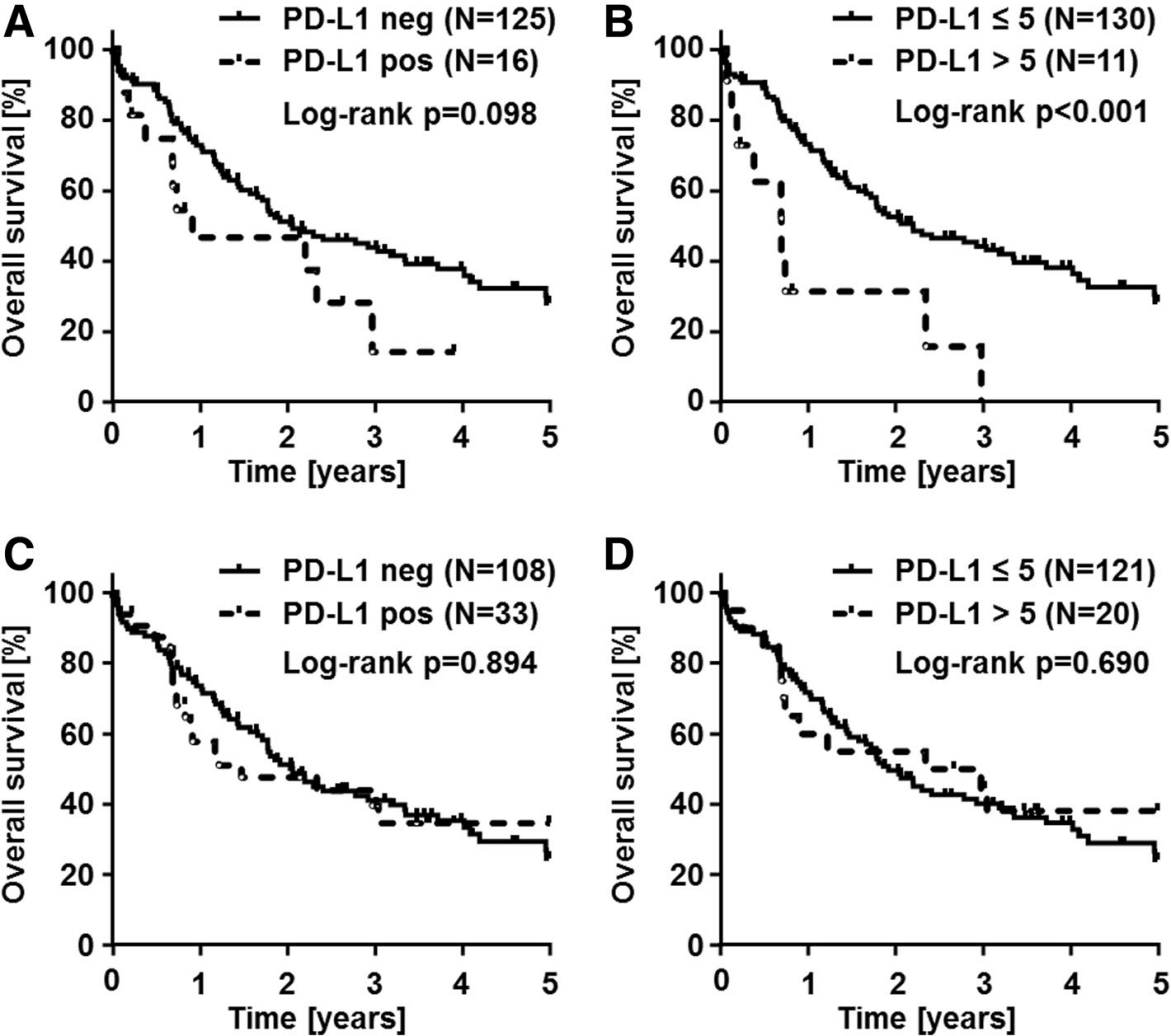
PD-L1 status in correlation with overall survival in cholangiocarcinoma patients. PD-L1 immunohistochemistry results using clone SP263 were correlated with overall survival of CCA patients. Kaplan-Meier curves show a trend of decreased overall survival in CCA patients with any PD-L1 positivity in tumor cells (*p = 0.09;* **a**). Significant decreased overall survival rates are seen in CCA patients with PD-L1 positivity in > 5% of tumor cells (*p < 0.001*; **b**). PD-L1 positivity in stromal cells has no impact on overall survival of CCA patients in none vs any (*p = 0.89;* **c**) or testing the cut-off of 5% (*p = 0.69;* **d**)

**Fig. 3:**
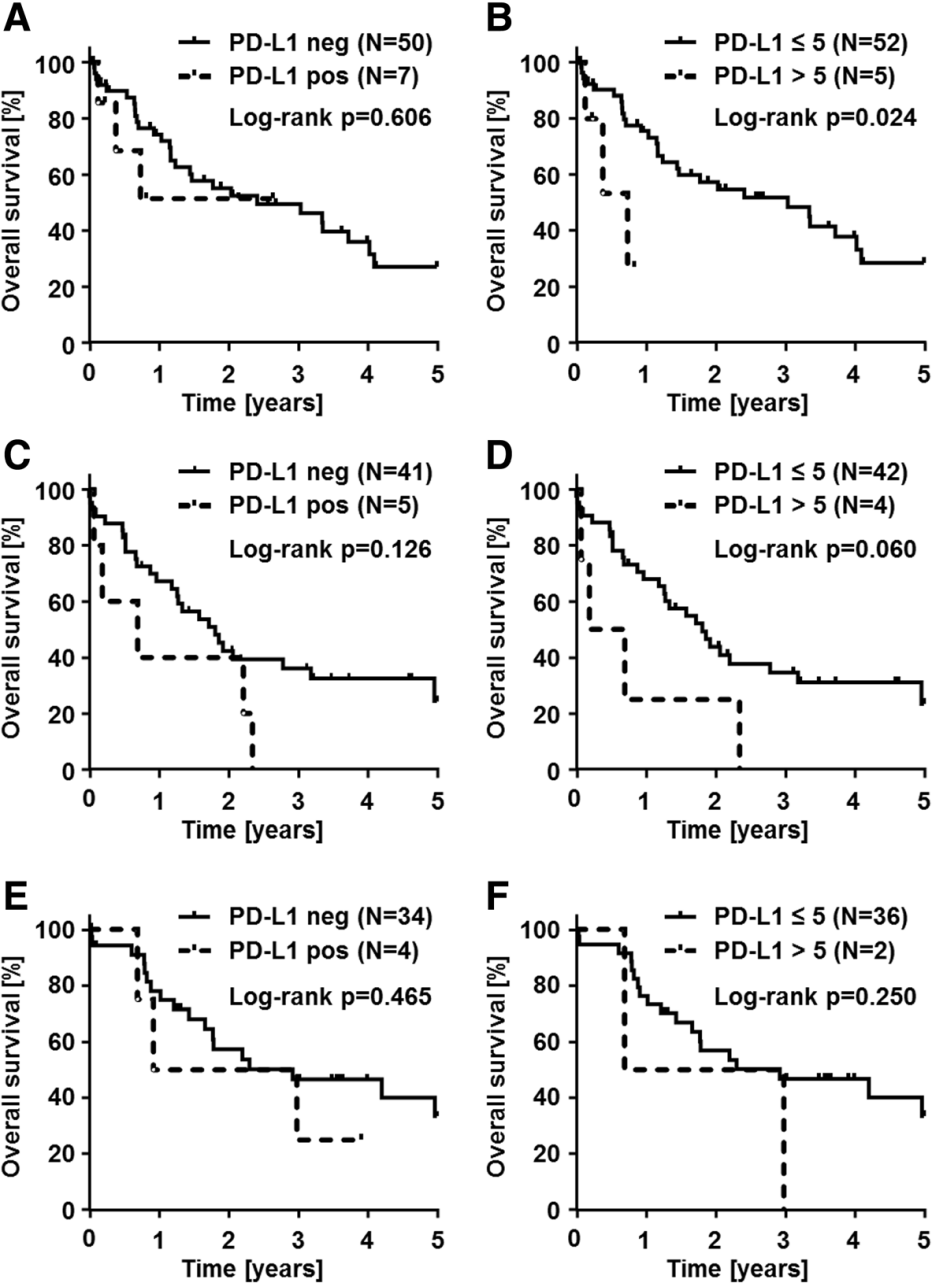
PD-L1 status in correlation with patient overall survival in cholangiocarcinoma subtypes. PD-L1 immunohistochemistry (clone SP263) results are correlated with overall survival of CCA patients, stratified by CCA subtypes. In intrahepatic CCA (iCCA), Kaplan-Meier curves show no significant overall patient survival difference in correlation with none vs any PD-L1 positivity in tumor cells (**a**), while significant decreased overall survival rates are seen in iCCA patients with PD-L1 positivity in > 5% of tumor cells (*p = 0.024)*; **(b)**). In perihilar CCA (pCCA), Kaplan-Meier curves show no significant overall patient survival difference in correlation with none vs any PD-L1 positivity in tumor cells (**c**), while by trend decreased overall survival rates are in pCCA patients with PD-L1 positivity in > 5% of tumor cells (*p = 0.060*; **(d**)). PD-L1 positivity in distal CCA (dCCA) has no impact on overall patient survival in none vs any (**e**) or testing the cut-off of 5% (**f**)

Correlation analyses with clinicopathological data including patient survival were conducted based on the staining results of the antibody with the highest rate of positivity (clone SP263). Overall survival data and PD-L1 staining was available for 141 patients. Testing different cut-offs of PD-L1 expression in association with patient survival showed differences by trend in none vs any PD-L1 expression, and even more significant results using the cut-off of > 5% PD-L1-positive tumor cells, while all other pre-selected cut-offs were not statistically significant (Additional file 2: Table S1). PD-L1 expression in > 5% of tumor cells was associated with shortened patient overall survival *p < 0.001*), whereas stromal PD-L1 expression was not associated with any difference in patient survival (*p = 0.89*) (Fig. 2). Stratification of clinicopathological variables using this cut-off is displayed in detail in Table 1. CCA subtype specific analysis for PD-L1 expression in association with patient survival showed decreased overall survival rates in iCCA patients with PD-L1 positivity in > 5% of tumor cells (*p = 0.02,* Fig. 3b) and decreased overall survival rates by trend in pCCA patients with PD-L1 positivity in > 5% of tumor cells (*p = 0.06,* Fig. 3d). PD-L1 expression in stroma cells was not associated with differences in survival of CCA patients. Correlation of PD-L1 status with all other clinicopathological data showed no significant association.

## Discussion

PD-L1 inhibitors have emerged as a novel and highly effective treatment option in a subset of cancer patients. Recent data suggests, that PD-L1 testing by immunohistochemistry prior to immunotherapy may be used to  select patients who will most likely respond to therapy with PD-L1 inhibitors [19, 20] and has therefore been approved as companion diagnostic tests for therapeutic anti-PD-1 antibodies in lung, kidney and urothelial carcinoma, as well as in malignant melanoma [6–8, 20]. As many aspects of PD-L1 testing in association with CCA have not been addressed systematically so far, we conducted this study i) to evaluate the expression levels of PD-L1 in CCA ii) to compare three different and commonly used antibodies against PD-L1 (clones 28–8, SP142 and SP263) and iii) to correlate PD-L1 expression levels with clinicopathological data, including overall survival.

A summary of the previous studies on PD-L1 expression in CCA that are PubMed-listed is highlighted in Table 4. The results obtained in these studies are highly heterogeneous, ranging from 4 -100% PD-L1 positive cases in iCCA, and 8 -  43% PD-L1 positive cases in extrahepatic CCA, including pCCA and dCCA. To date, the largest single investigation comprises tissue samples of 99 CCA patients [21]. While earlier studies reported relatively high positivity rates, newer investigations could not confirm these high PD-L1 positivity rates in CCA. In our study, a total of 170 cancer samples, including 72 iCCAs, 57 pCCAs and 41 dCCAs were analyzed using a systematic tissue microarray approach. In agreement with the studies from 2017, we here report PD-L1 positivity in CCA tumor cells in 5%, 4%, and 3% of iCCA, pCCA and dCCA, respectively, which is in line with the study of Sato et al. and among the lowest rates published to date. We hypothesize that the low rate of positive cases may be due to the different anti-PD-L1 clones and evaluation procedures used in the other studies. Furthermore, a smaller histological tumor area as a consequence of the usage of TMA could have contributed to the comparably low PD-L1 positivity rate in this study. We employed TMAs in order to standardize the staining procedure as much as possible among all cases. Inherent to the use of TMAs, there is always a possible selection bias, as it is not entirely clear if the extracted cancer regions are representative of the whole tumor for each given test. In this regard, it is especially important to note that PD-L1 is often only focally positive and that a positive staining has been described to be more likely at the invasion front of the tumor [22]. We tried to avoid this shortcoming by punching ≥4 cores of different tumor sites of each CCA to assure the best possible representation for each tumor. However, a bias resulting from the usage of TMAs cannot entirely be excluded.

**Table 4:** Meta-analysis of PubMed listed studies about PD-L1 immunohistochemistry in cholangiocarcinoma

Study	*n*=	Subtype	PD-L1 clone	Inflammatory cells, n/n (%)	Tumor cells, n/n (%)	Cut-off	Evaluation criteria
Ye et al., 2009	31	iCCA	28–8	n.a.	31/31 (100)	H-score^a^, no cut-off described	cytoplasmatic + membranous
Gani et al., 2016	54	iCCA	5H1	31/54 (57)	39/54 (72)	% positive cells (> 5%)	membranous
Sabbatino et al., 2016	27	iCCA	22C3	27/27 (100)	8/27 (30)	H-score^a^ (> 25)	membranous
Sato et al., 2017	68	iCCA, eCCA	28–8	n.a.	1/23 (4) 4/45 (8)	% positive cells (< 5, > 5, > 10%)	n.a.
Ma et al., 2017	70	eCCA	EPR1161	37/70 (53)	30/70 (43)	Immunohistochemical Score*: > 3	cytoplasmatic + membranous
Fontugne et al., 2017	99	iCCA, pCCA	E1L3N	31/58 (53) 15/41 (37)	5/58 (9) 4/41 (9)	Strong staining in > 5% of tumor cells	membranous
Sangkhamanon et al., 2017	46	n.a.	5H1	n.a.	32/46 (70)	> 1% positive tumor cells	membranous
Walter et al., 2017	69	pCCA dCCA	E1L3N	12/40 (30) 9/29 (31)	4/40 (10) 4/29 (14)	H-score^a^ (> 3)	membranous

*dCCA* distal cholangiocarcinoma, *eCCA* extrahepatic cholangiocarcinoma, *iCCA* intrahepatic cholangiocarcinoma, *pCCA* perihilar cholangiocarcinoma.^a^ Immunohistochemical scoring system: percentage of cells with weak staining × 1 + percentage of cells with moderate staining × 2 + percentage of cells with strong staining × 3 = H-Score *Immunohistochemical scoring system: Stained cells (0:< 5%,1:5–25%, 2:26–50%,3:> 50%) x intensity (1+, 2+, 3+) , n.a., not available

The differences in the percentage of positive samples can potentially be explained by the usage of different PD-L1 clones among the studies mentioned. The impact of using different PD-L1 clones is underlined by a recent study from the „Blueprint PD-L1 IHC Assay Comparison Project“, which demonstrated that the clones 28–8, 22C3 and SP263 show a similar proportion of positive tumor cells, whereas SP142 stains a lower proportion of tumor cells [10, 23]. In this study, we could confirm these findings for CCA, as with the clone SP142 only two cases were positive, while using the clones 28–8 and SP263, 18, respectively, 19 tumors were positive. While the results obtained with clone 28–8 and SP263 showed moderate agreement (*κ* = 0.44, *p* < 0.01), the agreement of 28–8 with SP142, and of 28–8 with SP263 were rather poor (*κ* = 0.13 and *κ* = 0.11, *p* < 0.01). However, this result could be partly caused by heterogeneity of PD-L1 expression which cannot be accounted for by the use of TMAs in this study. Moreover, due to the fact that in most of the reported studies, clones were used that were not included in our PD-L1 antibody panel, it is difficult to compare the given results. Furthermore, the PD-L1 antibody clones used in most of the reported studies were also not included in a current German PD-L1 harmonization study and are therefore not currently advised for routine use in Germany [10]. Nevertheless, other antibody clones should be tested in order to find the appropriate clone(s) to reliably detect patients that will benefit from anti-PD-L1 therapy.

Up to now, it has generally been accepted in immunohistochemical PD-L1 testing that only membranous and not cytoplasmic staining should be considered [10]. Of the previous studies on PD-L1 testing in CCA (Table 4), only five studies restricted their evaluation on membranous staining of the tumor cells, and in one investigation no comment about the mode of evaluation was made [21, 22, 24–28]. Thus, the results obtained by *Ye* et al. and *Ma* et al. cannot be compared to the other studies, including our own. It is also currently recommended to score only the proportion of positive cells, regardless of their intensity, as it became evident that this represents the only relevant parameter for predicting response to anti-PD-L1 therapy [10]. Interestingly, the amount of positive tumor cells seems to correlate with response to therapy in other cancer types [19, 29]. Some authors use the H-score, which consists of multiplication of intensity and percentage of positive cells. This approach is debatable, as the H-score is probably not an ideal scoring system for PD-L1 positivity. For example, ≥25% cells stained with low intensity and ≥ 9% of cells stained with strong intensity would classify for overall positivity based on the H-score. Compared to many clinical trials which require only ≥1% of tumor cells to have membranous staining for positivity, the H-score defines a comparably high cut-off. Regardless, the immunohistochemical scores that include staining intensity have been applied in a substantial number of studies of our meta-analysis in Table 4. As we set our cut-off to ≥1% positive tumor cells, one would expect the percentage of positive cases in our study to be higher than in the other three investigations. However, we could not detect membranous PD-L1 positivity on tumor cells in the majority of CCA samples. Various clinicopathological variables have been associated with PD-L1 positivity, including venous invasion [25], nodal metastases [26], high tumor grade [25] and high clinical stage [28]. However, we could not confirm these findings in this study by correlating PD-L1 status with clinicopathological data of our CCA patients.

In a recent meta-analysis involving more than 16,000 patients from 97 eligible studies on solid tumors, it was shown that PD-L1 expression assessed by immunohistochemistry significantly correlated with decreased overall and disease-free survival rates of patients [17]. This could be due to the fact that high PD-L1 levels have been shown to reduce T-cell function and to weaken the host’s immune response against the tumor [1]. In this regard, previous studies demonstrated the association of high PD-L1 expression and decreased survival rates  also in CCA [22, 24, 26]. However, other investigators could not confirm this finding  [25, 28]. Our data corrobates the findings of PD-L1 associated decreased overall survival in CCA patients. Additionally, using a PD-L1 expression cut-off of > 5% tumor cells, PD-L1 expression displays some prognostic stratification power in CCA patients (*p < 0.001*; Fig. 2b). Notably, prognostic significance was only detected using the cut-off of 5% of PD-L1 positive tumor cells. Whether PD-L1 positive CCAs show better response to anti-PD-L1 treatment and whether one clone is superior to reliably identify these patients is not clear based on the available data. This question should be addressed in future clinical trials. We did not detect a prognostic impact of PD-L1 positive stromal cells. However, detailed characterization of PD-L1 positive stromal cells merit future investigations. In addition, the question of whether specific stromal cells may predict the response to novel therapeutic approaches represents an interesting task for future functional studies of the stromal compartment in CCA.

## Conclusions

Here, we report in a large and well-characterized cohort of non-liver fluke associated CCAs that the frequency of PD-L1 positive CCAs is low, employing stringent immunohistochemical PD-L1 testing guidelines. Apart from an association of decreased overall survival in patients with  PD-L1 positive tumors, no significant association of PD-L1 status with any other clinicopathological variable of CCA patients was detected. Additionally, we showed that the selection of appropriate PD-L1 antibodies and careful evaluation of immunohistochemical staining patterns have a great impact on PD-L1 testing in CCA. We hope that our study paves the way for clinical trials that are clearly required to investigate the effects of PD-L1 targeted immunotherapy in CCA patients.

## Additional files

Additional file 1: **Figure S1.** Example of a typical staining pattern of on-slide control tissues (PD-L1, SP263). Tonsillary crypt epithelium showed strong membranous immunoreactivity (black triangle), while immune cells in germinal centers were weakly positive (black arrow, A). Gallbladder epithelium (black triangle) was negative but occasionally few immune cells were positive (black arrow, B). (PPTX 2692 kb)

Additional file 2: **Table S1.** Clinicopathological characteristics of the CCA cohort and comparison of patients with PD-L1 positive tumor cells in ≤5% or > 5%. A detailed overview on the clinicopathological characteristics of CCA exhibiting ≤5% or > 5% PD-L1 positive tumor cells is given. (XLSX 11 kb)

## Abbreviations

CCA: Cholangiocarcinoma
dCCA: Distal cholangiocarcinoma
iCCA: Intrahepatic cholangiocarcinoma
NCT: National Center for Tumor Diseases
pCCA: Perihilar cholangiocarcinoma
PD-L1: Programmed cell death ligand 1
TMA: Tissue microarray
WHO: World Health Organization
