# Supplementary figures and images for: Programmed cell death ligand 1 (PD-L1, CD274) in cholangiocarcinoma – correlation with clinicopathological data and comparison of antibodies
Source: BMC Cancer. 2019 Jan 15;19:72. doi: 10.1186/s12885-018-5254-0 (PMC6332835; doi:10.1186/s12885-018-5254-0)

## Slide 1
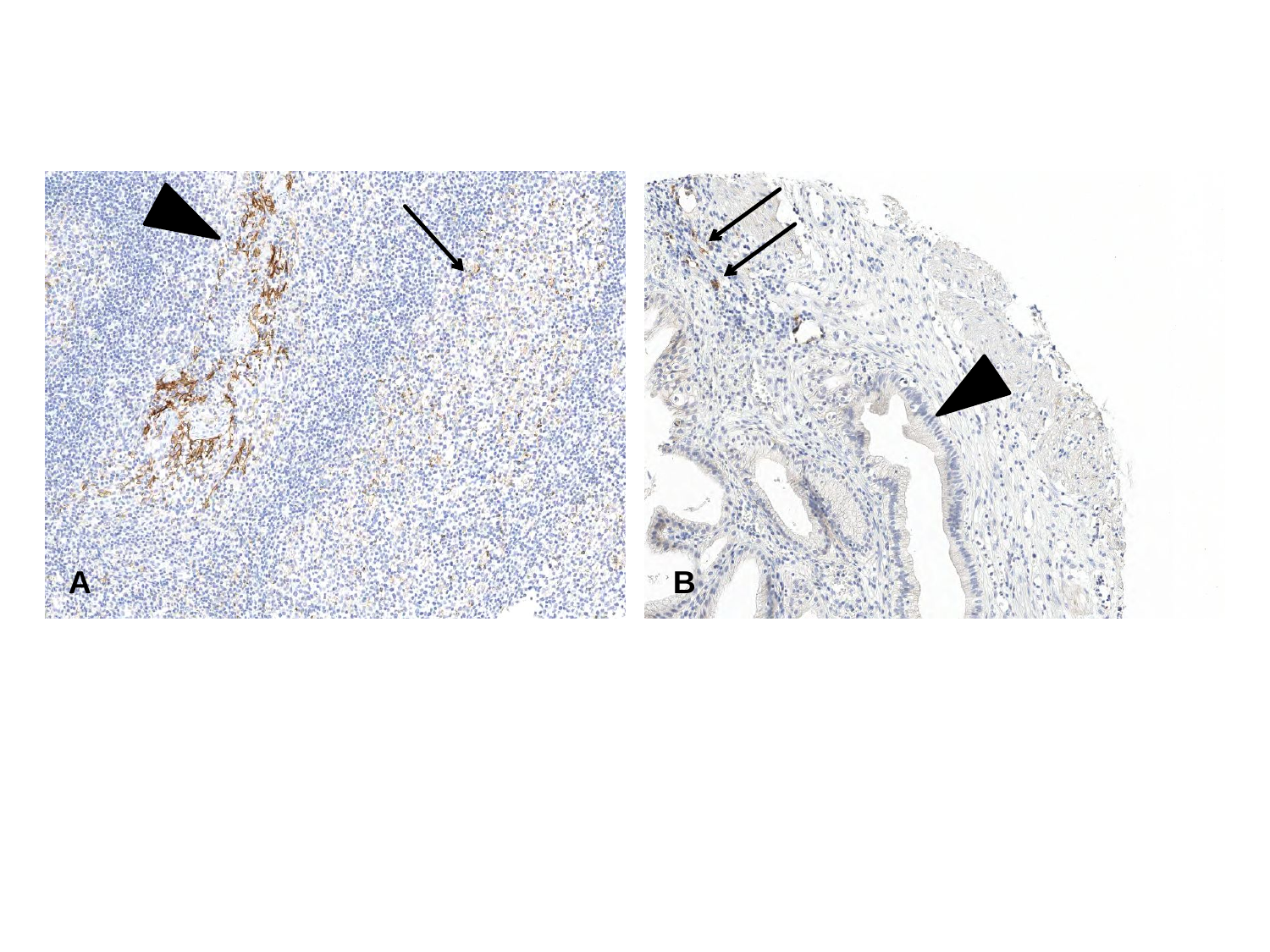

A
B

Supplement: Supplementary file 1 — Figure S1. Example of a typical staining pattern of on-slide control tissues (PD-L1, SP263). Tonsillary crypt epithelium showed strong membranous immunoreactivity (black triangle), while immune cells in germinal centers were weakly positive (black arrow, A). Gallbladder epithelium (black triangle) was negative but occasionally few immune cells were positive (black arrow, B). (PPTX 2692 kb) [file 12885_2018_5254_MOESM1_ESM.pptx]
